# Factors associated with common mental disorders among female nursing professionals in primary health care

**DOI:** 10.1186/s41155-018-0101-4

**Published:** 2018-07-25

**Authors:** Iracema Lua, Tânia Maria de Araújo, Kionna Oliveira Bernardes Santos, Maura Maria Guimarães de Almeida

**Affiliations:** 1 Universidade Estadual de Feira de Santana, Avenida Transnordestina, s/n, Bairro Novo Horizonte, Feira de Santana, BA 44036-900 Brazil; 2 Universidade Federal da Bahia, Salvador, Brazil

**Keywords:** Mental disorders, Female professionals, Nursing, Workers’ health, Primary health care

## Abstract

**Objective:**

To analyze the factors associated with common mental disorders (CMD) in primary care female nursing professionals.

**Methods:**

We performed exploratory cross-sectional study, using a structured questionnaire, applied to 451 primary care female nursing professionals from five municipalities of Bahia, Brazil, in a randomly selected sample. The outcome variable (CMD) was evaluated by SRQ-20. To analyze the factors associated with the prevalence of CMD, logistic regression analysis was used in blocks.

**Results:**

The prevalence of CMD was 16.2% and the exposure factors were professional category (PR 0.56, CI 0.34–0.90, *p* = .01), having a workload of more than 60 h per week (PR 2.53, CI 1.55–4.11, *p* < .01), personal insecurity at work (PR 1.92, CI 1.28–2.88, *p* = .00), high domestic overload (PR 1.94, CI 1.25–2.98, *p* < .01), effort-reward imbalance at work (PR 1.78, CI 0.98–3.23, *p* = .05), dissatisfaction with oneself (PR 2.30, CI 1.52–3.46, *p* < .01), poor quality of life (PR 1.69, CI 1.07–2.65, *p* = .02), and negative health status self-assessment (PR 1.77, CI 1.12–2.77, *p* = .01).

**Conclusions:**

The results reinforce the evidences of the relation between the health-disease process and work, be it professional or domestic. They also highlight the importance of changes in the organization of the nursing activities in the context of primary care, aiming to minimize stress and occupational dissatisfaction and promote the health of this category. It is noteworthy that domestic work should be considered and incorporated into investigations regarding the mental health of female population.

## Background

Common mental disorders (CMD) are characterized by insomnia, fatigue, irritability, forgetfulness, difficulty concentrating, and somatic complaints (Goldberg and Huxley, 1993). These disorders develop when the reaction of the subject opposite to the characteristics of the processes and work environments results in a disruption of the normal functioning of the individual, generating feelings of unworthiness and uselessness (Araújo, Pinho, and Almeida, 2005) and mental distress situations (Dejours, 1992). CMD represent the fourth leading cause of disability worldwide (Carvalho, Melo-Filho, Carvalho, and Amorim, 2013). Responsible for intense suffering, absenteeism, and work abandonment. They also are harmful to the daily activities of the individual, damaging their social and physical functions. Thus, they contribute to high public spending, becoming a public health problem (Braga, Carvalho, and Binder, 2010).

In addition, these disorders also affect the reinsertion of workers into new jobs. In case of reintegration processes, the disorders’ impact is even higher, which makes the work characteristics more precarious. Studies in Europe point out that people with a mental disorder have a higher risk of job loss and permanent exclusion from the job market, with 55% of the people with mental health problems making unsuccessful attempts to return to work, and from those who return, 68% have less responsibility, work less hours and receive lower wages than before (Organization for Economic Co-operation and Development [OECD], 2012).

In Brazil, CMD are even more relevant when analyzing epidemiological data on mental health disorders. We do not have, for example, statistics on the real situation, either in general populations or in working populations, and are thus dealing with problem that are still underestimated (Araújo, Palma, and Araújo, 2017). The health sector stands out among the occupational groups with the greatest mental illness, along with occupations such as teaching, banking, and informal workers. High prevalence of mental disorders among service workers has been consistently observed (Cordeiro, Mattos, Cardoso, Santos, and Araújo, 2016).

Therefore, mental disorders have been highlighted as one of the most important contemporary problems of public health, with a significant growth process worldwide, reaching different occupational groups (Cordeiro et al. 2016). A study on construction workers in a cohort of Dutch masons and supervisors recorded mental disorders in 27% of the employees (Boschman, Van Der Molen, Frings-Dresen, and Sluiter, 2014); a cross-sectional study found a prevalence of mental disorders of 44.4% in hospital cleaning professionals in Egypt (Abbas, Hammam, El-Gohary, Sabik, and Hunter, 2013); while in England it reached 14.1% of the workers (Stansfeld et al. 2013). This last study was part of a national research program on mental health in Britain, evaluating different professions—among health workers, a 17.9% prevalence of CMD was identified. In Brazil, mental and behavioral disorders constitute the third cause of illness-aid (Silva Júnior and Fischer, 2015). In the health sector, which integrates the large service sector, it is remarked that the rate of these types of disorders is relevant.

The evaluation of mental disorders in health workers has evidenced their occurrence in different contexts: a registering of mental disorders in 14% of the nurses from two teaching hospitals in Sydney, Australia (Perry, Lamont, Brunero, Gallagher, and Duffield, 2015); 42% of work-related mental disorders, 29% depression, and 24% anxiety, in Dutch hospital doctors (Ruitenburg, Frings-Dresen, and Sluiter, 2012). In Brazil, a high prevalence of CMD in health workers is observed, ranging in 10.3% in anesthesiologists in Belo Horizonte, MG (Neves and Pinheiro, 2012) to 48.6% in Community Health Agents of Pelotas, RS (Knuth et al. 2015). A CMD prevalence of 21% among healthcare workers in the Primary Care in Salvador (BA) (Oliveira and Araújo, 2018) and of 25% among female health workers in the Primary Care of Feira de Santana (BA) (Carvalho, Araujo, and Bernardes, 2016) is also worth noting.

Among health workers, nursing is classified as the fourth most stressful profession (Murofuse, Abranches, and Napoleão, 2005). It has, in its essence, the care for the patient who is suffering and at risk of death. It is a profession with significant emotional exhaustion and experiences of occupational stress situations. Most studies involving mental health and work is conducted in hospital settings, with the CMD prevalence ranging from 14.6% in a specialized hospital in Bahia (Souza, Martins Júnior, Silva, Costa, and Sobrinho, 2011) to 44.6%, in a hospital in São Paulo (Baptista and Tito, 2014).

Numerous situations have been identified as being associated with CMD among nursing workers, including individual, social, and working factors; among them are domestic overload (Araújo, Aquino, Menezes, Santos, and Aguiar, 2003) and the psychosocial aspects of work, evaluated by the demand-control model of Karasek (Araújo et al. 2003; Kirchhof et al. 2009; Pinho and Araújo, 2007) or the effort-reward imbalance model (Souza, Carvalho, Araújo, and Porto, 2011).

As already mentioned, most of the studies found in the literature have as setting the hospital network (Araújo et al. 2003; Arruda, 2014; Baptista and Tito, 2014; Kirchhof et al. 2009; Pinho and Araújo, 2007; Rodrigues, Rodrigues, Oliveira, Laudano, and Sobrinho, 2014; Souza, Martins Júnior, et al. 2011). Few studies have evaluated nursing workers in primary care. In this context, the overall analysis of workers without further specification of specific professional groups has been dominant (Araújo, Mattos, Almeida, and Santos, 2016; Braga et al. 2010; Carvalho et al. 2016; Tomasi et al. 2008). Among these workers, those of the nursing sector were found to be among the five health professions with higher prevalence of CMD (48% in nurses and 43.8% in nursing assistants) (Braga et al. 2010). Thus, a more specific and in-depth approach of this professional group is necessary, both in terms of the mental illness that has been identified and its importance for the actions and services of primary health care as for the illness proper.

Healthcare in Brazil occurs by the means of health services organized in a network, in a hierarchical and decentralized manner, with primary care as a gateway. In this model, emphasis is placed on the use of light technologies, such as bonding, reception, and monitoring the population (Brasil, 2012). Despite the advances and redefinition of the healthcare model, there are important difficulties in the work process and the conditions under which it is performed, which prevent or reduce the scope of the actions carried out, since they are not only limited to the diagnosis or treatment orientation. Added to the insalubrity of these services is the direct contact with pathogens and working in areas at risk of violence in the urban peripheries, resulting in dissatisfaction, exhaustion, high turnover, diseases, and occupational accidents.

A cross-sectional study with female primary care nursing professionals of a municipality of Bahia reported a prevalence of 28.6% of high emotional exhaustion and 46.4% reduction of professional fulfillment (Merces et al. 2016). This study also showed that primary health care professionals (physicians and nurses) in the municipality of Havana, Cuba, had higher rates of occupational burnout and stress symptoms when compared to hospital professionals (Hernández, 2003). These results reinforce the need to investigate the repercussions of the conditions and characteristics of nursing work in primary health care, in order to guide intervention programs to prevent these diseases and promote the health of this collective of workers, as well as the quality of the provided service.

In addition to the negative impacts that mental illness brings to the affected person, their family, and their work, there is evidence of the association of mental illness with general errors and errors in the performed procedures, such as medication, which have direct consequences on patient safety and satisfaction (Gärtner, Nieuwenhuijsen, Van Dijk, and Sluiter, 2010).

As described, there is consistent evidence of the high mental illness level among health workers in general and among nursing staff. Thus, it is important to analyze the factors that favor or determine this illness. This study is anchored in the perspective of collective health, guided by the paradigm of health surveillance, whose focus is the search for more effective responses to health needs, having as a presupposition the integral vision of the health-disease process, involving the concepts of vulnerability and determination of health, with emphasis on the factors and/or causes towards the promotion of health (Brasil, 2017).

The focus of this study, therefore, is to evaluate conditions and characteristics associated with mental disorders in female nursing professionals. Despite the wide range of analyzed factors, the emphasis of this study was focused on occupational factors, seeking to evaluate the contributions of this study. Appropriate work conditions can prevent or reduce illness and have a positive impact on job maintenance. Therefore, intervening in working conditions/characteristics may have an impact on absenteeism, productivity, frequency of disabilities, and mental illness. In this perspective, this study aimed to analyze the factors associated with common mental disorders in female primary care nursing professionals, with special emphasis on occupational aspects.

## Methods

It is an exploratory, cross-sectional epidemiological study carried out with data from the multicenter project “Working Conditions, Conditions of Employment and Health of Health Workers,” developed by the Núcleo de Epidemiologia of the Universidade Estadual de Feira de Santana (UEFS).

The study population was made up of female nursing professionals from the primary health care services of five municipalities in Bahia: Feira de Santana, Jequié, Itabuna, Santo Antônio de Jesus, and the sanitary district of the Historical Center of Salvador. The selection was made by random sampling stratified by geographic area, defined by the areas covered by the Núcleo de Apoio à Saúde da Família (NASFs in Portuguese) and occupational group. This study focused on nursing professionals. In addition, it was decided to analyze only women considering (a) higher prevalence of CMD are described among women (Araújo et al. 2003); (b) small number of men in the study population (in this study only seven men); (c) existence of differences in the working conditions between men and women (lower recognition posts with more unfavorable general characteristics among women, even though it is a predominantly female profession). The definition of restricting the analysis only to women was defined based on these factors.

As the sample for this study was not designed to estimate the events of interest, specifically in female nursing professionals, the sample estimates were redefined, based on the formula for a finite population, taking the following as a parameter: total population of 909 female primary care nursing professionals in the cities studied, 95% confidence level, absolute accuracy of 3%, and the expected proportion of 16% CMD (Tomasi et al. 2008), resulting in a sample size of 352, plus 20% to compensate for possible losses and refusals, the sample size was stipulated in 422 female workers.

Data collection took place in the years 2011–2012, using a self-administered questionnaire, based on previous studies with health workers (Pinho and Araújo, 2007) and tested in a pilot study with 30 workers from a municipality in Bahia not participating in this study. Furthermore, for some dimensions analyzed, instruments already validated in Brazil have been used, such as psychosocial aspects of work (occupational stressors), measured by the Job Content Questionnaire (JCQ) (Araújo and Karasek, 2008; Santos, Araújo, Carvalho, and Karasek, 2017; Santos, Carvalho, and Araújo, 2016b) and by the effort reward imbalance (ERI) (Chor, Werneck, Faerstein, Alves, and Rotenberg, 2008; Silva and Barreto, 2010).

The JCQ evaluates aspects related to social and psychological, and it may be applied to several types of occupational contexts. In this study, its version translated into Portuguese and validated by Araújo and Karasek (2008) was used. A recent study (Santos et al. 2016b) confirmed the multifactorial structure and good adjustment of the questionnaire, as well as its appropriate internal consistency (Cronbach alpha and composite reliability). The recommended version includes 49 Likert-type questions with four scales (1 = strongly disagree to 4 = strongly agree), and these questions were organized in five dimensions: (1) control over work, (2) physical and (3) psychological demand; (4) social support, and (5) personal insecurity at work. In order to develop those indicators, the variables of each proposed scale were summed, taking into consideration the weighting factors expected to the model. The demand (low/high) and the control (low/high) were dichotomized by using the average as a cut-off point (as recommended by JCQ guide).

The ERI is used to study imbalance situations between effort and reward at work. The structure of the answers was similar to JCQ, with consistency validated in Norway, Belgium, Japan, Finland, and Germany. In Brazil, some validation studies were conducted with 111 health workers, and employees of a university (Chor et al. 2008) and 100 bank employees (Silva and Barreto, 2010), and they showed great performed (Cronbach alpha higher than 0.70 for all scales).

The outcome variable (CMD) was assessed by the Self-Reporting Questionnaire (SRQ-20), an instrument for mental illness screening (Harding et al. 1980), validated for use in Brazil in a population of workers in Bahia (Santos, Araújo, Pinho, and Silva, 2010; Santos, Carvalho, and Araújo, 2016a), presenting satisfactory performance indicators. The instrument consists of 20 questions with dichotomous answers (0 = no and 1 = yes), computing 1 point for each positive answer. In this study, the cut-off point for CMD screening was adopted for seven or more positive responses—recommended as the best cut-off point for women by Santos et al. (2010).

The variables of exposure were categorized and grouped into blocks of analysis: sociodemographic variables, life habits, characteristics and conditions of work, psychosocial aspects of work, job satisfaction, and quality of life and health situation (Table 1).

**Table 1:** Exposure variables and their categories of analysis

Original variables	Categorized variables for analyses
Sociodemographic variables	
Age (continuous variable)	0 = up to 40 years 1 = more than 40 years
Race/skin color	0 = white 1 = non-white (yellow, brown, indigenous, black)
Marital status	0 = with partner (consensual union/stable union, married) 1 = no partner (single, divorced/separated/widowed)
Presence of children	0 = no 1 = yes
Life habits	
Practice of leisure activities	0 = yes 1 = no
Physical activity practice	0 = yes (1 to 2 times a week, 3 or more times a week) 1 = no (never)
Use of alcoholic beverages	0 = no 1 = yes
Smoking	0 = non-smoker (non-smoker, former smoker) 1 = current smoker
Job characteristics and conditions	
Professional category (open question)	0 = nurse 1 = nursing assistant/technician
Working time (continuous variable)	0 = less than 5 year 1 = 5 to 10 years 2 = more than 10 years
Working time at the unit (continuous variable)	0 = less than 5 year 1 = 5 to 10 years 2 = more than 10 years
Employment bond	0 = full employment (municipal with public service exam-of the permanent staff contracted by CLT) 1 = precarious work (municipality or assigned worker to the state or federal government; service provider; cooperative worker; trustee position; outsourced; trainee)
Having another employment bond	0 = no (I have no other work) Yes, at the municipality; yes, at the state; yes, I have another job in the private sector with a formal contract; yes, I have another job on my own; yes, in another municipality; yes, I have another job in the private sector without a formal contract)
Having labor rights (do you have the right to the 13th salary, holidays, vacations, and the additional holiday wages)	0 = yes, all (they had all the labor rights) 1 = yes, partially (they had 1 to 3 of the rights) 2 = no (they had none of the labor rights)
Weekly working week (continuous variable)	0 = up to 40 h 1 = between 41 and 60 h 2 = more than 60 h
Work shift	0 = daytime (morning, afternoon, morning, and afternoon) 1 = night work/work in shifts
Physical demand on the work	0 = low physical demand (lower than the average sum) 1 = high physical demand (higher than the average sum)
Compatibility of the developed activities	0 = compatible with the position (yes, totally; yes, most of the time) 1 = not compatible with the position (yes, most of the time; almost never; never)
Personal safety threatened at work	0 = no 1 = yes
Domestic work	0 = no 1 = yes
Household overload: sum of basic household tasks (washing, ironing, cleaning, cooking), evaluated by the number of dwellers, by the formula: HO = (washing + ironing + cleaning + cooking) × (M − 1) (Aquino, 1996), categorized as tertiles: high, medium, and low overload, as used by Pinho and Araújo (2012) and then dichotomized.	0 = low/medium overload 1 = high overload
Psychosocial aspects of work	
Social support	-High support (higher than average) -Low support (below average)
Control demand model: construction of four categories of work, from the combination of the dimensions psychological demand and control over work (JCQ guide).	0 = low demand (high control and low demand) 1 = active work (high control and high demand) 2 = passive work (low control and low demand) 3 = high demand (low control and high demand)
Reward-effort model (ERI): formula ER = effort/(reward × 0.5454) (correction factor).	0 = balance (result of function < 1) 1 = imbalance (result of function ≥1)
Work satisfaction and quality of life	
Satisfaction with working ability	0 = satisfied (satisfied; very satisfied) 1 = dissatisfied (very dissatisfied; dissatisfied; neither dissatisfied nor satisfied)
Work satisfaction	0 = satisfied (I am satisfied, I am very satisfied) 1 = unsatisfied (I am not satisfied at all; I’m not satisfied)
Satisfaction with salary/income (question “Taking into account all my efforts and achievements, is my salary/income adequate”)	0 = satisfied (agree and strongly agree) 1 = dissatisfied (disagree, strongly disagree)
Satisfaction with personal relationships	0 = satisfied (satisfied; very satisfied) 1 = dissatisfied (very dissatisfied; dissatisfied; neither dissatisfied nor satisfied)
Satisfaction with oneself	0 = satisfied (satisfied; very satisfied) 1 = dissatisfied (very dissatisfied; dissatisfied; neither dissatisfied nor satisfied)
Quality of life assessment	0 = Good (good; very good) 1 = Poor (very poor; poor, neither poor nor good)
Health situation	
Occupational or occupational diseases	-No -Yes
Accidents at work	-No -Yes
Medical license	-No -Yes
Presence of comorbidity: sum of 26 variables related to diseases diagnosed by a physician, all with two response categories (1 = yes, 0 = no)	-No (absence of comorbidity) -Yes (presence of comorbidities for any positive response)
Presence of musculoskeletal pain: sum of the variables related to this dimension: pain in the legs, pain in the arms, pain in the upper back, and pain in the lower part of the back, all with five categories of responses (0 = never, 1 = rarely, 2 = infrequent, 3 = frequent and 4 = very frequent). Initially, these variables were dichotomized in no (0) for “never” and “rarely” and in yes (1) for the other categories, then being summed.	0 = no (all negative responses) 1 = yes (any positive response)
Self-assessment of health status	0 = positive (very good/good) 1 = negative (regular/poor/very poor)

Univariate, bivariate, and multivariate analysis were performed in blocks. Absolute and relative frequencies were estimated for the characterization of the sample. In the bivariate analysis, the prevalence ratios (PR) were calculated. Pearson’s chi-square test or Fisher’s exact test were used to identify statistical significance, using a *p* ≤ 0.25 value for the preselection of variables to be included in the multivariate analysis, respecting previous theoretical criteria.

In the multivariate analysis, we used logistic regression in blocks, *Backward* method of selection, using *dummy* variables for those with more than two categories. The analysis was performed in the following two steps: (I) block-by-block modeling, analysis of the RP adjusted in each block, using as selection criterion for the following step the value of *p* ≤ 0.17 (Hosmer and Lemeshow, 2000); (II) interblock modeling, gradual adjustment of the variables of the six blocks of analysis, considering the following order of entry: sociodemographic, life habits, work characteristics (general characteristics and psychosocial aspects), work satisfaction, and quality of life and health situation. In the final model remained the factors associated with CMD at a 5% significance level.

In the final model, the prevalence ratios were estimated using Poisson’s regression with robust variance. The adequacy of this model was evaluated by the goodness of fit test (Hosmer and Lemeshow), area under the ROC curve, and analysis of the patterns of influential observations and collinearity between the variables of the final model.

Data typing and processing were performed in the *Statistic Package for Social Sciences*-SPSS 24.0 for *Windows* and data analysis in the *STATA for Windows* version 7.0 program.

The multicenter study was Approved the by the Ethics in Research Committee of the UEFS (Protocol 081/2009; CAAE0086.0.059.000-091) on 30 November 2009. The informed consent form was signed by all participants.

## Results

A total of 451 female primary health care nursing professionals were interviewed. Four hundred thirty-eight of them responded to the SRQ-20 (loss of 2.8%, 13 workers). As the data loss was low, the minimum sample number (*n* = 422) was obtained, even computing the losses (*n* = 438). The comparative analysis between the characteristics of responders and non-responders to the SRQ-20 showed that there were no significant differences between them, reducing the possibility of selection bias due to data loss. The data analysis was performed without imputation of values.

As for sociodemographic characteristics, 63.1% were up to 40 years old, 79.1% were from race/color non-white, 52.6% had a partner, and 60.9% had children. In terms of lifestyle, the majority of these nursing professionals performed regular leisure activities (83.8%) did not practice physical activities (61.6%), did not consume alcoholic drinks (59.4%), and did not smoke (96.6%) (Table 2).

**Table 2:** Prevalence of common mental disorders according to sociodemographic variables and lifestyle habits

Variables (*N*)	*n* (%)	Common mental disorders		
		*n* (P%)	PR	*p* value
Sociodemographic				
Age (444)*				
Up to 40 years	280 (63.1)	45 (16.2)	1.00	
40 or more	164 (36.9)	26 (16.6)	1.02	.93
Race/skin color (445)*				
White	93 (20.9)	12 (13.5)	1.00	
Non-white	352 (79.1)	58 (16.9)	1.25	.44
Marital status (449)*				
With partner	236 (52.6)	41 (18.0)	1.00	
Without partner	213 (47.4)	30 (14.4)	0.80	.31
Children (448)*				
No	175 (39.1)	28 (16.4)	1.00	
Yes	273 (60.9)	43 (16.3)	0.99	.98
Lifestyle habits				
Leisure activity practice (445)*				
Yes	373 (83.8)	48 (13.0)	1.00	
No	72 (16.2)	23 (34.8)	2.68	< .01
Physical activity practice (401)*				
Yes	154 (38.4)	15 (9.9)	1.00	
No	247 (61.6)	37 (15.5)	1.57	.11
Alcohol consumption (394)*				
No	234 (59.4)	32 (14.2)	1.00	
Yes	160 (40.6)	26 (16.8)	1.18	.48
Smoking (443)*				
Non-smoking	428 (96.6)	67 (16.0)	1.00	
Current smoker	15 (3.4)	1 (8.3)	0.52	.82**

*Variables with missing values

**Fisher’s exact test

The prevalence of CMD was 16.2%. None of the analyzed sociodemographic variables were statistically associated with CMD, being selected for multivariate analysis by theoretical criteria. As for lifestyle habits, leisure and physical activity were associated with CMD, and selected for multivariate analysis.

As predominant job characteristics, we highlighted the following: being a nursing technician/assistant (66.7%), workload and less than 5 years worktime in the unit (38.2 and 67.4%, respectively), outsourced work (52.9%), having only one employment bond (63.8%), partial labor rights (71.2%), a workday of up to 40 h (70.1%), daytime labor (83.3%), involving low physical demand at work (56.2%), performing activities that were compatible with their positions (95.1%), not feeling their personal safety threatened at work (62%), performing domestic activities (54%), and low to medium domestic overload (61.8%) (Table 3).

**Table 3:** Common mental disorders prevalence according to employment status and psychosocial aspects of the job

Variables (*N*)	*n* (%)	Common mental disorders		
		*n* (P%)	PR	*p* value
Working characteristics and conditions				
Professional category (451)				
Nurse	150 (33.3)	30 (20.1)	1.00	
Nursing technician/assistant	301 (66.7)	41 (14.2)	0.71	.10
Worktime (448)*				
Less than 5 years	171 (38.2)	19 (11.2)	1.00	
Between 5 and 10 years	109 (24.3)	19 (17.8)	1.59	.12
Longer than 10 years	168 (37.5)	33 (20.8)	1.86	.01
Worktime in the unit (448)*				
Less than 5 years	302 (67.4)	42 (14.2)	1.00	
Between 5 and 10 years	95 (21.2)	17 (18.9)	1.33	.27
Longer than 10 years	51 (11.4)	11 (22.4)	1.58	.13
Employment bond (446)*				
Employment stability	210 (47.1)	40 (19.9)	1.00	
Outsourced work	236 (52.9)	31 (13.4)	0.67	.06
Having another employment bond (450)*				
No	287 (63.8)	36 (12.9)	1.00	
Yes	163 (36.2)	35 (22.2)	1.72	.01
Having labor rights (441)*				
Yes, all	101 (22.9)	16 (16.7)	1.00	
Yes, partially	314 (71.2)	46 (15.0)	0.90	.77
No, none	26 (5.9)	5 (19.2)	1.15	.87**
Weekly work week (435)*				
Up to 40 h	305 (70.1)	38 (12.8)	1.00	
Between 40 and 60 h	79 (18.2)	14 (18.2)	1.42	.22
More than 60 h	51 (11.7)	18 (36.0)	2.81	< .01
Working shift (449)*				
Daytime	374 (83.3)	63 (17.1)	1.00	
Nightshift/24 h shift	75 (16.7)	7 (10.4)	0.61	.17
Physical demand at work (447)*				
Low physical demand	251 (56.2)	33 (13.5)	1.00	
High physical demand	196 (43.8)	38 (19.9)	1.47	.07
Activities that were compatible with their position (449)*				
Yes	427 (95.1)	68 (16.4)	1.00	
No	22 (4.9)	2 (9.5)	0.58	.63**
Personal safety threatened at work (450)*				
No	279 (62.0)	31 (11.4)	1.00	
Yes	171 (38.0)	40 (24.0)	2.10	< .01
Domestic work (448)*				
No	204 (45.5)	26 (13.0)	1.00	
Yes	244 (54.5)	45 (19.1)	1.47	.08
Domestic overload (440)*				
Low/medium overload	272 (61.8)	33 (12.5)	1.00	
High overload	168 (38.2)	36 (22.1)	1.77	< .01
Psychosocial aspects of the job				
Social support (423)*				
High support	225 (53.2)	24 (11.0)	1.00	
Low support	198 (46.8)	40 (20.6)	1.87	< .01
Demand-control model (423)*				
Low demand jobs	136 (32.2)	13 (9.6)	1.00	
Active jobs	101 (23.9)	18 (18.6)	1.94	.04
Passive jobs	100 (23.6)	19 (19.6)	2.04	.03
High demand jobs	86 (20.3)	19 (22.4)	2.33	< .01
Effort-reward imbalance model (436)*				
Balance	140 (32.1)	12 (8.8)	1.00	
Imbalance	296 (67.9)	58 (20.1)	2.28	< .01

*Variables with missing values;

**Fisher’s exact test

Among these variables, the ones that showed positive and statistically significant associations with CMD were worktime between 5 and 10 years (PR 1.59, *p* = .12) and over 10 years (PR 1.86; *p* = .01), worktime in the unit longer than 10 years (PR 1.58, *p* = .13), workload per week between 40 and 60 h (PR 1.42, *p* = .22) and greater than 60 h (PR 2.81, *p* < .01), personal safety at work (PR 2.10, *p* < .01), physical demand during work (PR 1.47; *p* = .07), domestic work (PR 1.47; *p* = .08), and domestic overload (PR 1.77; *p* < .01). The ones that showed a significant negative association with CMD were professional category (PR 0.71, *p* = .10), employment bond (PR 0.67, *p* = .06), working shift (PR 0.61, *p* = .17). All these variables were selected for multivariate analysis (Table 3).

A high number of the female nursing professionals stated they received little social support at work (46.8%). According to the demand-control model (DCM), 43.9% experienced unfavorable work situations (20.3% in high-demand work and 23.6% in passive work). The effort-reward imbalance model (ERI) identified a high percentage of the professionals experiencing imbalance between the effort made in their work activities and the received rewards (67.9%). All the variables analyzed in this block were positively associated to CMD (Table 3), being included in the multivariate model.

The data in Table 4 show that the female nursing professionals were satisfied with their working ability (78.4%), with their work (75.5%), with personal relationships (86.9%) with themselves (84.9%), and had a good quality of life (71.1%). All variables in this block met the criteria previously established and were selected for the multivariate analysis.

**Table 4:** Common mental disorders prevalence according to job satisfaction and quality of life

Job satisfaction and quality of life (*N*)	*n* (%)	Common mental disorders		
		*n* (P%)	PR	*p* value
Working ability (450)*				
Satisfied	353 (78.4)	42 (12.2)	1.00	
Dissatisfied	97 (21.6)	29 (30.9)	2.53	< .01
Satisfaction with their work (449)*				
Satisfied	339 (75.5)	35 (10.7)	1.00	
Dissatisfied	110 (24.5)	35 (32.4)	3.03	< .01
Satisfaction with salary/income (447)*				
Satisfied	75 (16.8)	14 (18.7)	1.00	
Dissatisfied	372 (83.2)	57 (15.8)	0.85	.54
Satisfaction with personal relationships (450)*				
Satisfied	391 (86.9)	47 (12.3)	1.00	
Dissatisfied	59 (13.1)	23 (41.8)	3.39	< .01
Satisfaction with oneself (451)				
Satisfied	383 (84.9)	46 (12.4)	1.00	
Dissatisfied	68 (15.1)	25 (37.3)	3.01	< .01
Evaluation of quality of life (450)*				
Good	320 (71.1)	31 (10.0)	1.00	
Bad	130 (28.9)	40 (31.7)	3.17	< .01

*Variables with missing values

The investigation of the health situation revealed that 7.9% had an occupational or professional illness diagnosed; 5.4% suffered accidents at work or on the way to it; 34.1% obtained medical leave or were dismissed from work in the last 12 months; 75.3% had some type of comorbidity; 65.2% reported musculoskeletal pain; and 15.8% self-perceived their health status negatively. When analyzing the associations with CMD, statistical significance was only not observed in the medical leave variable, which was excluded from the multivariate analysis (Table 5).

**Table 5:** Common mental disorders prevalence according to the health status of the female nursing professionals

Health status	*n* (%)	Common mental disorders		
		*n* (P%)	PR	*p* value
Occupational or professional illness (444)*				
No	409 (92.1)	57 (14.2)	1.00	
Yes	35 (7.9)	13 (39.4)	2.77	< .01
Accidents at work (445)*				
No	421 (94.5)	64 (15.6)	1.00	
Yes	24 (5.4)	7 (29.2)	1.87	.15**
Medical leave (449)*				
No	296 (65.9)	44 (15.2)	1.00	
Yes	153 (34.1)	27 (18.2)	1.19	.40
Presence of comorbidity (441)*				
No	109 (24.7)	8 (7.5)	1.00	
Yes	332 (75.3)	61 (18.7)	2.49	< .01
Presence of musculoskeletal pain (345)*				
No	120 (34.8)	5 (4.3)	1.00	
Yes	225 (65.2)	44 (20.1)	4.67	< .01
Self-perceived health status (450)*				
Positive	379 (84.2)	46 (12.5)	1.00	
Negative	71 (15.8)	25 (35.7)	2.86	< .01

*Variables with missing values;

**Fisher’s exact test

The final logistic regression model identified that professional category, having a weekly workload higher than 60 h, feeling personal security threatened at work, having a domestic work overload, experiencing effort-reward imbalance (ERI), having poor self-satisfaction, poor quality of life, and negative health status self-assessment, were associated with CMD (Table 6). Consequently, these variables were those that were associated with CMD in the study population, according to the adopted selection criteria.

**Table 6:** Factors associated with common mental disorders in nursing professionals, obtained through the multivariate analysis

Factors associated with CMD	PR _adjusted_	CI 95%	*p* value
Professional category			
Nursing technician/assistant	0.56	0.34–0.90	.01
Weekly work week			
More than 60 h	2.53	1.55–4.11	< .01
Personal safety threatened at work			
Yes	1.92	1.28–2.88	< .01
Domestic overload			
High overload	1.94	1.25–2.98	< .01
Effort-reward imbalance model			
Imbalance	1.78	0.98–3.23	.05
Satisfaction with themselves			
Dissatisfied	2.30	1.52–3.46	< .01
Quality of life evaluation			
Bad	1.69	1.07–2.65	.02
Self-perceived health status			
Negative	1.77	1.12–2.77	.01

It should be stressed that, according to the theory (Abbas et al. 2013; Farias and Araújo, 2011; Pinho and Araújo, 2012), being a nurse (variable “professional category”) was considered reference category (unexposed) for data analysis, however, the obtained PR (< 1.0) showed the opposite situation, in which the nurses were more exposed to mental illness than nursing technician.

The evaluation of the final model revealed that it was adequate (Hosmer and Lemeshow goodness of fit = 0.537), fitted well to the data (area under the ROC curve = 0.82), and did not have influential data or colinearity among the variables.

## Discussion

The prevalence of CMD found (16.2%) was equivalent to the one observed among primary care health professionals of the Brazilian Northeast and South (16%) (Tomasi et al. 2008). However, a study in primary care units in Botucatu (São Paulo, Brazil) found higher prevalence (48% for nurses and 43.8% for nursing technicians/assistants) (Braga et al. 2010). This variation may be associated with the employment characteristics in the investigated contexts. In this study, temporary work predominated, as has been commonly reported, as the predominant health employment profile in the northeast region. Such precariousness of the nursing profession can influence the provided answers: instability in the employment bond can produce a positive response bias due to the constant fear of unemployment. It is also possible that continuous hiring and firing mechanisms are operating, in which only the healthiest workers maintain their jobs.

Brazilian studies with nursing professionals from the hospital network found distinct CMD prevalence: in the northeast, the prevalence ranged from 14.6 (Souza, Martins Júnior, et al. 2011) to 35% (Rodrigues et al. 2014.); in the south and southeast, prevalence rates from 18.7 (Kirchhof et al. 2009) to 44.6% (Baptista and Tito, 2014) were observed. The observed variations allow us to infer that regional iniquities imply differences in the environment and working conditions, specifically affecting the workers’ mental health conditions. It is worth mentioning that although all the aforementioned studies used the same research instrument (SRQ-20), cut-off points for suspected CMD were not presented. Thus, it is possible that this definition influenced the estimated prevalence, compromising the compatibility among the studies. Therefore, it is necessary to standardize the forms of analysis by means of a validation study of this instrument for this professional category, in order to obtain a better comparison.

The factors associated with CMD were professional category, longer working hours, personal insecurity at work, effort-reward imbalance, high domestic overload, dissatisfaction with oneself, poor quality of life, and negative self-assessment of health. This reveals an association between the working, domestic and professional, and sickness causing characteristics of these female professionals, as well as between satisfaction with life and work and health conditions of the individual.

Evidence of increased exposure to mental illness by female nurses diverges from the understanding that individuals with higher schooling are less exposed to illness (Abbas et al. 2013; Farias and Araújo, 2011; Pinho and Araújo, 2012). It seems that in nursing, this relationship is reversed: a cross-sectional study with workers from Botucatu’s primary health network (São Paulo, Brazil) showed a 9.5% increase in CMD among nurses (Braga et al. 2010); another investigation in two hospitals in Manaus (Amazonas, Brazil) found a 13% higher CMD prevalence among nurses (Arruda, 2014); in the hospital network of Bahia (Brazil), nurses had a CMD prevalence 23.7% higher when compared to the auxiliaries and 8% higher than the nursing technicians (Rodrigues et al. 2014). This reality is also identified in specialized hospital professionals (Feira de Santana, Bahia, Brazil), with 8% more CMD among nurses (Souza, Martins Júnior, et al. 2011). Only a study with female nursing professionals from a public hospital in Salvador (Bahia, Brazil) showed evidence of a higher prevalence of CMD among nursing technicians (36.4%, compared to 20% in nurses) (Araújo et al. 2003).

This higher evidence of exposure to mental illness among nurses can be attributed to their work process, in which multiple tasks are developed, including management and care. This entails an intense rhythm, activity accumulation, and work overload. These characteristics are not exclusive of hospital work as they can also be seen in primary care (Almeida, 2012). Due to this overload, the job invades the nurse’s “way of life,” extending the workday and often preventing the development of quality work.

We identified that the workload per day was associated with mental illness among the female nursing professionals, corroborating the findings in the literature that identify a 33% higher prevalence of CMD among female urban workers with longer working hours (Farias and Araújo, 2011); in primary health care workers, this difference was 92% (Oliveira, 2013). Long workdays are common among health professionals, as a result of multiple employment bonds, which in turn are a reflection of low wages and employment insecurity, due to the precariousness of health work in Brazil. The long hours dedicated to work produce two events that together can potentiate the negative effects of work: (a) prolonged alertness, which increases the production of the so-called stress hormones (Karasek and Theorell, 1990), requiring that the individuals should be prepared to meet the demands of work—in activities that involve risks for others, such as health activities, the pressure for attention, and vigilance increases even more; (b) reduction of the time for activities of resting, physical activity, and leisure constitute mechanisms of recovery and physical and mental balance. Thus, the greater the workload, the more vulnerable will the situation for mental health be.

The workload per day of the studied professionals is incorporated into the domestic work, exposing them to the two working environments which entails physical and psychic overload (Araújo et al. 2005). A study with workers from the urban area of Feira de Santana (Bahia, Brazil) showed a CMD prevalence 5.23 times higher among domestic service workers when compared to the reference category (transportation sector) (Farias and Araújo, 2011). Therefore, when analyzing the workload, especially in occupations predominantly composed of women, such as health and education, one must also include the analysis of the contribution of the domestic workload (Araújo et al. 2005; Farias and Araújo, 2011). In this way, the necessary visibility of health of the women exposed to this double working day will be attained.

Female workers with high domestic overload had almost twice as many CMD compared to women with a low/medium overload. In addition to the overload attributed to double working hours, women are still subject to the devaluation of women’s work, considered an important triggering factor for physical and mental exhaustion (Araújo et al. 2005).

The imbalance between the efforts made at work and the received reward was also identified as a factor associated with mental illness. Similar results have been observed in other studies in the health sector, with primary care health workers (Oliveira, 2013) and hospital nursing professionals (Arruda, 2014), and in other sectors, such as high-voltage network electricians (Souza, Carvalho, et al. 2011). In these studies, robust and consistent associations between situations of imbalance between efforts and rewards at work and mental illness were observed. Thus, aspects related to the perception of justice, of balance between what is given and what is received at work also plays, as observed, a relevant role in the psychic balance. The perception that what is being given does not match what is being achieved seems to produce feelings of devaluation and non-recognition, generating suffering. The continuity of this imbalance is an open door to mental illness.

Hence, the hypothesis that the non-balancing of effort and rewards at work can be harmful to mental health (Siegrist, 1996) was strengthened. The characteristics of the working process in nursing evidence this situation as the work involves high effort and low rewards, attributed to lagged salaries and lack of recognition by co-workers and users.

In primary care, recognition comes from the community, and it is necessary to build a bond for the continuity and integrality of this care. In the Brazilian context, for many of the actions, the workers do not have total governability and depend on the support of the Municipal Health Secretaries and subsequent levels of attention belonging to the Health Care Networks.

This support is often precarious, compromising the quality of the provided assistance and the users’ satisfaction. These situations, that compromise the recognition of the effort given by nursing professionals, end up contributing with feelings of inadequacy and uselessness which, in turn, produce psychic suffering (Almeida, 2012). A research in nursing primary health care workers in a municipality of Bahia identified high prevalence of moderate/high emotional exhaustion (61.6%), high depersonalization (48.3%) and low job satisfaction (56.6%) (Merces et al. 2017), which, in turn, produce physical illness and mental.

Job insecurity was another factor associated with CMD. Several studies have shown that direct contact with communities, especially suburban ones, may represent exposure to violence, establishing a relationship between the latter and mental illness (Assunção and Silva, 2013; Oliveira, 2013; Yang, Wong, and Coid, 2013).

In primary care, work activities are performed in the health units and in their coverage areas, which are often high-risk areas. In addition, these professionals, especially the nursing team, remain longer and in greater interaction with patients and their companions, being constantly exposed to violent acts. This can cause permanent worrying, favoring the development of emotional symptoms, such as frustration, stress, sadness, anger, discouragement, and low self-esteem (Gasparini, Barreto, and Assunção, 2006). The relationship between lack of safety in the work environment and mental illness of the worker is thus established.

The association between satisfaction and mental health situation is consistently observed (Haddad, 2000), showing that mental health is favored when the individual is in harmony with himself and his environment. The findings showed that personal dissatisfaction was associated with CMD. Satisfaction stems from an assessment that the duty has been fulfilled and that a given social utility was attained (the desires at work have been achieved) which cannot always be done in real working conditions. It is also noteworthy that satisfaction is an important indicator of resilience that can contribute to reduction of the impacts generated by the working conditions and environment in mental illness.

The negative evaluation of quality of life was also associated with CMD, corroborating a study of Jansen et al. (2011): higher prevalence rates of CMD related to the worst levels of quality of life. Poor quality of life can influence both the onset and permanence of mental disorders (Jansen et al. 2011), and may have coincidental factors, that is, working conditions and environments have direct impacts on quality of life and mental health (Alvarenga and Marchiori, 2014). Therefore, the findings confirm that quality of life and CMD are correlated in different ways.

Therefore, health is reaffirmed as a biopsychosocial well-being, in which physical and mental health are interdependent, as already pointed out in other studies (Abbas et al. 2013; Assunção and Silva, 2013; Jansen et al. 2011; Merces et al. 2016). The nursing professionals who negatively assessed their health status had a 77% higher prevalence of CMD than those who positively assessed their health status, which is similar to results from other studies (Arruda, 2014; Assunção and Silva, 2013). It is worth remembering that health self-assessment encompasses various aspects, involving a multidimensional structure which includes the mental health conditions, to the extent that this indicator considers the signs and symptoms of diseases and their impact on the physical, mental, and social well-being (Griep, Rotenberg, Landsbergis, and Vasconcellos-Silva, 2011).

The results found in this study indicated the importance of several factors in the mental illness of the individuals, with emphasis on working conditions. This reinforces the need for studies deepening the investigation of each of these specific factors in the occurrence of mental disorders in the nursing work activities as well as the real impacts of this illness on their life and work.

There is a clear need for investments to promote the mental health of health professionals, especially through the implementation of policies to promote and protect the workers’ health, aiming at raising the quality of life and satisfaction of this category, as well as the quality of care provided to the users of the Unified Health System (Gärtner et al. 2010).

There are some aspects that should be considered in the evaluation of the results of this study. Since it is a cross-sectional study, it is not possible to define the chronological order of the events, which makes it impossible to establish causal relationships. It is neither possible to rule out reverse causality, since worse health levels can lead to the overestimation of risk situations. Another important limitation of this study design is the survival bias and the effect of the healthy worker, as we only analyzed only employed individuals that present the effect of interest at the time of the research and remained working, which can underestimate the actual prevalence of the disease (prevalence bias).

Therefore, the need for future investigations, through longitudinal studies to diagnose the actual prevalence and/or incidence of CMD and the causal pathway of the associations identified with them, needs to be highlighted. The planning and programming of health actions based on Health Surveillance requires a detailed knowledge of the occurrence and understanding of the factors that determine the distribution of diseases related to health, in order to propose measures to address the health necessities of the workers.

Intervention studies and/or extension projects in partnership with the Reference Centers in Occupational Health (Centro de Referência em Saúde do Trabalhador-CEREST in Portuguese) are also relevant, as they would allow to recognize locally the risk factors (physical, chemical, biological, ergonomic, of accidents, and psychosocial) in the nursing work processes and environments. These actions may favor the shared planning coping strategies to increase the resilience of these workers—understood by Sousa and Araujo (2015) as a set of social and intrapsychic processes that enable a healthy life in an unfavorable environment. In addition, permanent educational actions should be developed to understand psychological diseases and ways to overcome stigmas of them. In case of sickening, the workers should be taken to the Network of Psychosocial Attention (Rede de Atenção Psicossocial-RAPS in Portuguese) of reference. This will allow the delineation of lines of care for the integral and equitable attention of the workers in psychological suffering, including therapeutic measures integrated with cultural aspects.

## Conclusions

We identified that being a nurse, having more than 60 h of workload per week, feeling personal safety threatened at work, high domestic overload, imbalance between work-rewards efforts, being dissatisfied with oneself, poor quality of life, and negatively self-rated health status, are factors associated with the occurrence of common mental disorders.

These findings show the relationship between the health-disease process and the living and working conditions, be it professional or domestic, as well as the association between physical and mental health. This knowledge can contribute to the formulation of strategies to cope with illness and promote and protect the workers’ health, based on Health Surveillance tools.

Despite the limitations of our study, the acquired information is useful and reaffirms the need for actions related to the promotion and protection of the health of health professionals, especially for female nursing professionals who work in direct contact with suffering, anguish, and death. It is worth noting that nursing professionals are extremely important for the implementation and consolidation of primary health care services, which are the basis of the health system. However, this will only become possible with healthy professionals who are satisfied with the work they perform.

We hope that our study will contribute to a reflection and a new critical look at the mental health of the female nursing professionals, giving visibility to the relations between gender, health, and work, including domestic work, so that this reality can be debated, rethought and reconstructed.

## Abbreviations

CEREST: Centro de Referência em Saúde do Trabalhador
CI: Confidence intervals
CMD: Common mental disorders
DCM: Demand-control model
ERI: Effort-reward imbalance
NASF: Núcleo de Apoio à Saúde da Família
OR: Odds ratio
P: Prevalence
PHC: Primary health care
PR: Prevalence ratios
RAPS: Rede de Atenção Psicossocial
ROC: Receiver operating characteristic
SPSS: Statistic Package for Social Sciences
SRQ-20: Self-Reporting Questionnaire-20
UEFS: Universidade Estadual de Feira de Santana
